# Ultrasound-assisted extraction and encapsulation of betalain from prickly pear: Process optimization, *in-vitro* digestive stability, and development of functional gummies

**DOI:** 10.1016/j.ultsonch.2024.106975

**Published:** 2024-06-27

**Authors:** Deepak Mehta, Kritika Kuksal, Kamlendra Yadav, Sudesh Kumar Yadav, Yuqin Zhang, Shivraj Hariram Nile

**Affiliations:** a Division of Food and Nutritional Biotechnology, National Agri-Food Biotechnology Institute (NABI), Sector-81, Mohali 140306, Punjab, India; b Institute of Himalayan Bioresource Technology, Palampur, Himachal Pradesh, 176061; c College of Pharmacy, Fujian University of Traditional Chinese Medicine, Fuzhou, Fujian, 350122, PR China

**Keywords:** Ultrasonication, Prickly pear, Natural pigments, Encapsulation, Shelf-life, Stability

## Abstract

The study aimed to extract and encapsulate betalain pigment from prickly pear (*Opuntia ficus*-indica) using ultrasound-assisted extraction and eco-friendly glycerol. Subsequent analysis encompassed assessing its thermal stability, shelf-life, bio-accessibility, and biological properties. The process optimization employed Response Surface Methodology (RSM), focusing on glycerol concentration (20–50 %), sample to solvent ratio (1:10–1:20), temperature (30–60 °C), and time (10–30 min). Optimal conditions were determined as 23.15 % glycerol, 1:10 sample to solvent ratio, 10.43 min treatment time, and 31.15 °C temperature. Under these conditions, betalain content reached 858.28 mg/L with a 93.76 % encapsulation efficiency. Thermal stability tests (80–180 °C; 30 & 60 min) showed degradation of betalain with higher temperatures and longer durations, affecting the visual aspect (ΔE) of the pigment. Encapsulated betalain exhibited favorable shelf stability, with optimal storage life of 404.27 days at 4 °C in amber conditions, compared to 271.99 days at 4 °C without amber, 141.92 days at 25 °C without amber, and 134.22 days at 25 °C with amber. Bio-accessibility of encapsulated betalain was significantly higher (2.05 ± 0.03 %) than conventionally extracted pigment (1.03 ± 0.09 %). The encapsulated pigment displayed strong anti-inflammatory properties in dosages of 2–20 µL, with no cytotoxic effects. Additionally, incorporation into gummies was successful and visually approved by sensory panellists. Glycerol proved to be a green encapsulating agent for betalain, offering high shelf life and bio-accessibility, making it suitable for food industry applications. The encapsulated pigment demonstrated robust thermal stability and shelf life, making it suitable for food industry applications. This study highlights glycerol’s potential as a sustainable alternative for natural pigment extraction.

## Highlights

• Ultrasound-assisted extraction demonstrated effective recovery of betalain compounds from prickly pears.
• Glycerol as green solvent was utilized for both extraction and encapsulation of betalain, providing a dual action.
• Compared to conventional methods, encapsulated betalain demonstrated twice the bio-accessibility.
• The encapsulated pigment displayed anti-inflammatory properties without any observed cytotoxicity.

## Graphical abstract

**Figure:**
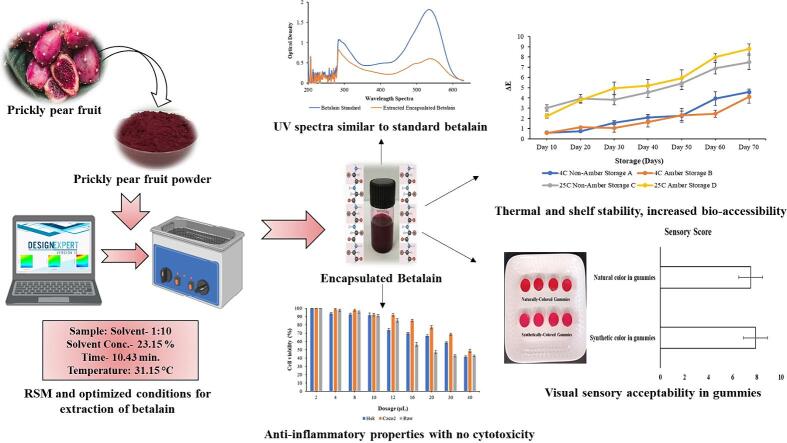

## 1 Introduction

Colour brings life to the world. Essentially, customers eat with their eyes, making colour one of the primary quality attributes in food products, alongside aroma, flavor, and freshness [1], [2]. Food colorants, added during processing or storage, significantly influence the visual appeal of food products. However, the use of artificial colours has come under scrutiny due to concerns about exceeding recommended doses, particularly among children [3]. Previous studies have linked overexposure to artificial colours with various health risks, including certain types of cancer, allergic reactions, digestive issues, and genotoxicity. These findings underscore the need for caution and further research into the potential health impacts of artificial food colorants and the search for natural alternatives [2], [4]. Considering the negative impacts of artificial colours, researchers and industries are exploring natural colour extracts that offer additional health or nutritional benefits. Natural colorants can be derived from plants, fruits, fruit or vegetable by-products, microorganisms, animals, and insects [5], [6]. These natural colours can be categorized according to their hue or chemical structure, such as flavonoids, anthocyanins, carotenoids, betalains, and chlorophylls. Among these, anthocyanins and betalains are particularly in demand due to their bright, attractive hues and water solubility, making them highly suitable for the food industry [6], [7].

Betalains are nitrogenous pigments derived from a tyrosine base and produced from betalamic acid. They can be further categorized into betacyanins and betaxanthins. Betacyanins are formed when betalamic acid condenses with *cyclo*-DOPA (3,4-dihydroxyphenylalanine) or its glucosyl derivatives, whereas betaxanthins are formed when betalamic acid conjugates with amino acids or their derivatives [7], [8]. Beetroot is the primary source of betalains, but other sources include species from the genera Amaranthus, Hylocereus, and Celosia. Additionally, Opuntia species, commonly known as prickly pear, are rich in betalains as well as other nutrients such as flavonoids, ascorbate, phenolic acids, soluble fibers, niacin, and carotenoids [9].

Prickly pear (*Opuntia ficus*-indica), an underutilized fruit, features a prickly outer layer, vibrant sweet flesh, and small seeds inside. Unlike other sources of betalains that do not tolerate extreme conditions well, prickly pear thrive in arid regions with minimal water and high temperatures [9], [10]. Belonging to the Cactaceae family, prickly pears are renowned for their unique nutritional and medicinal benefits. Research indicates that prickly pear extracts possess anti-ulcerogenic, neuroprotective, hepatoprotective, and antiproliferative properties [11]. The high nutrient content of prickly pear fruit is attributed to its ascorbic acid, phenolic compounds, betacyanins, and betaxanthins [10]. The fruit is composed of approximately 85 % water, 15 % sugar, 0.3 % ash, and less than 1 % protein, and is rich in minerals and amino acids such as asparagine, arginine, and alanine [12]. Given these benefits, prickly pear is an excellent source for extracting colour pigments like betacyanin and betaxanthin. There are several methods for extracting natural colour pigments from plant material, particularly from Opuntia fruits, such as Soxhlet extraction [13], solid–liquid extraction [14], and pressurized-liquid extraction [15]. Traditional extraction techniques often exhibit poor efficiency due to the side effects of oxidation and hydrolysis. Consequently, researchers have explored non-conventional procedures, such as microwave-assisted extraction and ultrasound-assisted extraction (UAE), to overcome these challenges [16], [17]. Ultrasound-assisted extraction is an eco-friendly and energy-efficient technology for extracting colour pigments. By utilizing ultrasonic waves, this method effectively disrupts cell walls, facilitating the release of intracellular compounds. This enhances mass transfer, allowing for a more efficient and thorough extraction process. Moreover, the accelerated extraction kinetics provided by ultrasound technology significantly reduce the time required for extraction. Due to these advantages, ultrasound-assisted extraction has gained widespread popularity and is increasingly employed across various industries. Additionally, ultrasound-assisted extraction can be easily integrated into existing production lines, offering scalability and adaptability. Its ability to operate under mild conditions helps preserve the bioactivity and stability of sensitive compounds, such as natural pigments, making it an ideal choice for extracting natural pigments and bioactive ingredients.

Researchers have explored ultrasound-assisted extraction technology for extracting phytochemicals from various plant materials. For instance, Brahmi et al. [18] reported the ultrasonic-based extraction of polyphenolic compounds from the flowers of *Opuntia ficus*-indica using an ethanol–water solvent system. Additionally, Melgar et al. [19] optimized the ultrasonication-assisted extraction conditions for polyphenolic compounds from the peel of *Opuntia engelmannii* cultivar. However, there are relatively few studies on the extraction and exploration of coloured pigments from the whole prickly pear fruit. Even fewer studies have investigated the ultrasonic-assisted extraction of colour pigments from whole prickly pear fruit using greener solvents like glycerol. The goal is to optimize these variables to enhance the extraction efficiency of betalains from freeze-dried prickly pear fruit powder. Furthermore, extracted colour pigments from prickly pear fruit have been evaluated for their thermal stability and shelf stability. In addition, the in-vitro digestibility and anti-inflammatory activity of these extracted colour pigments have also been assessed, with further applications in functional gummies. These investigations highlight the potential for using prickly pear fruit as a valuable source of natural colorants and bioactive compounds.

## 2 Materials and methods

### 2.1 Materials

Prickly pear fruit was procured from fruit and vegetable market, Sec-26, Chandigarh, India. Prickly pear fruit pulp and peel was grinded to form a paste like consistency, freeze dried at −80 °C, lyophilized to get fruit powder. Freeze-dried prickly pear powder was stored at 4 °C until further analysis. For gummies preparation, food grade ingredients like agar-agar, pectin, citric acid, table sugar, artificial color, gummy mould were procured from local market Sector-20, Chandigarh, India.

### 2.2 Chemicals

DPPH (2,2-diphenyl-1-picrylhydrazyl), fetal bovine serum (FBS), MTT, PBS, potassium chloride, monopotassium phosphate, sodium chloride, magnesium chloride hexahydrate, salivary amylase from human saliva, pepsin from porcine gastric mucosa, bile from bovine, pancreatin from porcine pancreas HPLC grade distilled water, methanol, glacial acetic acid and DMSO were procured from Sigma Aldrich (St. Louis, USA). Glycerol, sodium bicarbonate and ammonium carbonate were procured from Hi-Media Laboratories. Hydrochloric acid, calcium chloride dihydrate and sodium hydroxide were obtained from CDH. Human colorectal adenocarcinoma cells (Caco-2), Human embryonic kidney cells (HEK) and RAW 264.7 mouse macrophage cell lines were obtained from National Centre for Cell Science, Pune, India. DMEM and antibiotic (penistrep) were purchased from Gibco.

### 2.3 Experimental set-up

**Fig. 1:**
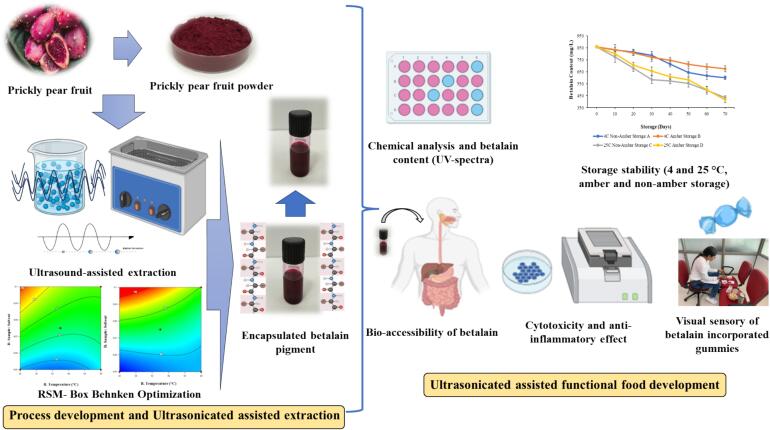
Workflow for optimized ultrasound assisted extraction of betalains, bio-accessibility of encapsulated extract, shelf stability and analyzing its cell viability and anti-inflammatory properties.

Initially, optimization for betalain (Betaxanthin + Betacyanin) extraction from freeze-dried prickly pear powder was conducted having different concentration of glycerol, solid/liquid ratio, time duration and extraction temperature. After getting optimized extraction conditions, samples were extracted accordingly and further centrifuged at 6000 rpm to get glycerol extract. This glycerol extract was further concentrated to get honey like consistency. This concentrated pigment was further analyzed for color value, betalain content, in-vitro digestibility, cell line studies and storage stability studies. Detail about work procedure was explained in flow chart (Fig. 1).

### 2.4 Optimization of betalain extraction from prickly pear powder

Optimization of extraction parameters for betalain (betaxanthin and betacyanin) was carried out using design of experiment (DoE) DX 12.0 of RSM. Prickly pear color pigment was extracted using aqueous glycerol and the extraction parameters were optimized using Box-Behnken design (BBD) in response surface methodology (RSM). Glycerol concentration (X1, 20–––50 %, w/v), solid/liquid ratio (X2, 1:10 to 1:20), time duration (X3, 10–30 min.) extraction temperature (X4, 30–60 °C) were chosen as independent variables, while Betaxanthin (Y1, mg/L), and Betacyanin (Y2, mg/L) were selected as the response variables. Each variable was coded at three levels − 1, 0 and 1 and a total of 29 randomized experimental runs were conducted (Table 1). The experimental data fitted to Quadratic model. Analysis of variance (ANOVA) was used to determine the significant difference of independent variables and their interactions, statistical significance of the model and the regression coefficients. After completion of extraction, samples were centrifuged at 6000 rpm to get glycerol extract, lyophilized to get concentrated betalain pigment and stored at 4 °C until further analysis.

**Table 1:** Color pigment response obtained under different conditions based on a Box-Behnken Design for response surface analysis. Values are reported as mean ± SD (n = 3).

**Sample run (n)**	**Time (min.)**	**Temperature (°C)**	**Solvent Concentration (%)**	**Sample: Solvent**	**Betaxanthin (mg/L)**	**Betacyanin (mg/L)**
1	30	30	35	0.075	72.38	144.28
2	10	45	20	0.075	84.32	170.13
3	30	45	35	0.1	78.41	294.25
4	20	45	35	0.075	64.17	141.35
5	10	45	35	0.1	83.55	283.25
6	20	45	35	0.075	65.45	158.03
7	20	30	35	0.1	77.51	150.52
8	20	45	35	0.075	67.38	146.3
9	30	45	50	0.075	55.83	132.18
10	30	45	20	0.075	61.09	138.23
11	20	45	20	0.1	129.62	307.08
12	30	60	35	0.075	63.14	110.73
13	20	30	35	0.05	36.25	64.63
14	20	60	35	0.1	87.01	166.83
15	30	45	35	0.05	36.83	92.58
16	20	45	35	0.075	74.56	163.9
17	20	45	50	0.1	223.94	467.5
18	20	60	50	0.075	69.17	126.31
19	10	30	35	0.075	96.89	178.93
20	20	45	20	0.05	31.83	84.15
21	20	45	35	0.075	56.08	139.7
22	20	45	50	0.05	20.34	46.2
23	20	60	20	0.075	62.75	122.38
24	10	45	35	0.05	40.11	78.74
25	20	30	50	0.075	90.22	161.88
26	20	60	35	0.05	46.71	84.33
27	10	60	35	0.075	72.5	138.6
28	20	30	20	0.075	82.13	152.9
29	10	45	50	0.075	63.39	132.73

### 2.5 Encapsulation efficiency

For the experimental analysis of betalain, 200 mg of sample was dispersed in 1 mL of a 50:8:42 v/v/v methanol: acetic acid: water mixture, vortexed for one min., ultrasonicated, centrifuged at 10,000 rpm for five min., and filtered using a 0.22 mm Millipore filter. For the analysis of surface betalain, 100 mg of sample were treated with 10 mL of ethanol: methanol (1:1), agitated for one min. in a vortex mixer, and then centrifuged for five min. at 10,000 rpm. Both version of samples was further quantified for the amounts of betalain using spectrophotometric instrument [20]. Encapsulation efficiency was calculated using following formula:

$$EncapsulationEfficiency\left(EE\right)\%:\frac{\mathrm{Experimentalbetalain}-Surfacebetalain}{\mathrm{Experimentalbetalain}}x100$$

### 2.6 Characterization of betalain content extracted from prickly pear

Betacyanin and betaxanthin content of the prickly pear extract were determined spectrophotometrically (Shimadzu, Milan, Italy) reported earlier [21]. The amount of betalains was computed using visible spectra (λ = 360–700 nm), the betacyanin was measured at 530 nm and betaxanthin at 480 nm and the analyses were performed in triplicate. Calculation of respective components given below:

$$BetacyaninorBetaxanthincontent\left(\frac{mg}{L}\right):\frac{\mathrm{AxDFxMW}}{\varepsilon xL}x1000$$

Where, A = absorbance, DF = dilution factor, MW = molecular weight (For betacyanin- 550 g/mol; For betaxanthin- 308 g/mol), ∊ = molar extinction coefficients (For betacyanin- 60,000 L/mol; For betaxanthin- 48,000 L/mol), and L = path length (1 cm).

### 2.7 Color measurement of encapsulated color pigment

Using a Hunter Lab colorimeter (Color-Flex EZ, Hunter Lab, Virginia, USA), the color parameters (L*, a*, and b*) of extracted color pigment were determined in triplicate [22]. The color parameters were calculated using the Hunter Lab colorimeter, where L* represented lightness and a* and b* represented red+/green- and yellow+/blue-, respectively. The outcomes were also shown as a hue angle and color difference.

$$HueAngle:\tan^{-1}\frac{b*}{a*}$$

$${ColorDifference(\Delta E}^{*}):\sqrt{{\left(L_{2}^{*}-L_{1}^{*}\right)}^{2}+{\left(a_{2}^{*}-a_{1}^{*}\right)}^{2}+{\left(b_{2}^{*}-b_{1}^{*}\right)}^{2}}$$

### 2.8 Thermal studies of encapsulated color pigment

To determine the influence of temperature on encapsulated betalain, they were exposed to different temperatures ranging from 80 to 160 °C for 30 and 60 min., respectively, and variations in betalain content, hue angle and color difference were analyzed [22]. The thermal degradation kinetics of extracted betalain pigment has been reported to follow first order reaction adequately [23]. The first order kinetics model based on betalain concentration is:

$$\ln\left(\frac{L}{\mathrm{Lo}}\right)=-kt$$

where, L represents final concentration, Lo represents initial concentration of betalain content and t represents duration of treatment time. Half-time was also calculated respectively based on rate constant of first order kinetics.

### 2.9 Stability of extracted and encapsulated color pigment against storage and light

According to Nascimento Filho et al. [24], the stability of encapsulated betalain content was investigated across a range of conditions, including amber, non-amber containment, and storage temperature (4 °C and 25 °C). The betalain content of encapsulated betalains was measured every 10 days for a maximum of 70 days. The findings were computed and presented as a percentage of betalain retention, and color difference as explained in 2.5, 2.6.

### 2.10 Predictive shelf-life assessment of encapsulated color pigment

After knowing and confirming first-order kinetics, the predictive shelf-life of betalain color pigment was calculated. The equation for predicting the shelf-life of color pigment is as follows:

$$t_{s}=\frac{\ln\left(\frac{N_{0}}{N_{t}}\right)}{k_{T}}$$

where, t_s_ is the predictive storage time, N_0_ is the content of betalain in the beginning of the shelf-life, N_t_ is assumed to be 25 % retention of betalain content from initial value in the beginning of shelf-life and k_T_ is the rate constant (k) at the storage time temperature T. The assumption of 20–25 % betalain retention in color pigment was determined on the basis studies published earlier [21], [25].

### 2.11 In-vitro digestibility of encapsulated color pigment

In this method, *in-vitro* digestibility of encapsulated color pigment extracted using current method was compared with conventionally extracted color pigment, to observe the effect of encapsulation. Conventional color pigment extraction was performed using method given by Saénz et al. [26]. *In-vitro* digestion was proceeded with the method mentioned by Brodkorb et al. [27] with some minor modifications. This digestion method consisted of three digestion phases- oral, gastric, and intestinal phase. Briefly, the oral phase was initiated by adding 1 g of extracted pigment in 5 mL of salivary simulated fluid (consisted of alpha-amylase and salts), mixed properly and pH was raised to 7.0. This reaction mixture was incubated in shaking incubator at rpm for 2 min. at 37 °C. The oral bolus from previous phase was taken and 10 mL of simulated gastric fluid (contain pepsin enzyme and salts) was added and pH was reduced to 3.0. This reaction mixture was incubated in shaking incubator for 2 h at 37 °C. The gastric chyme was added to 20 mL of simulated intestinal fluid (pancreatin, bile and salts) and pH was raised to 7.0. Incubated for 2 h in shaking incubator at 37 °C. After intestinal digestion, the temperature was raised to 100 °C for 4 min. to inactivate the enzyme. The supernatant of digestion mixture was collected by centrifugation at 4500 rpm for 20 min. at 4 °C and proceeded for analysis.

$$Bioaccessibility:\frac{\mathrm{Betalaincontentafterdigestion}}{\mathrm{Betalaincontentbeforedigestion}}x100$$

### 2.12 Cytotoxicity and anti-inflammatory properties of encapsulated color pigment

Human colorectal adenocarcinoma cells (Caco-2), Human embryonic kidney cells (HEK) and RAW 264.7 mouse macrophage cell lines were obtained from National Centre for Cell Science, Pune, India. Cells were cultured in DMEM media supplemented with 10 % (v/v) fetal bovine serum (FBS) and 1 %, v/v, antibiotics (100 U/mL penicillin and 100  μg/mL streptomycin) at 37 °C in a CO_2_ incubator at 5 % CO_2_. Cell viability was accessed with 3-(4,5-dimethythiazol-2-yl)-2,5-diphenyltetrazolium bromide (MTT) assay. Before experiment cells were seeded in 96 well plate at a density of 5x10^4^/mL, on reaching 80–90 % confluency cells were treated with encapsulated color betalain (1000 mg/mL) at concentration of 2–40 µL. After 24 h of exposer 10  μL MTT (5  mg/mL) was added to each well and plate was incubated for another 4  h in CO_2_ incubator. Further the cell culture media was removed and 100  μL of dimethyl sulphoxide (DMSO) was added to dissolve the formazan crystals and absorbance was recorded at 570  nm using a micro-plate reader (Spectra Max M5e, Molecular Devices, Minnesota, and USA). The cell viability was calculated using following formula:

$$CellViability\left(\%\right):\frac{A_{s}}{A_{c}}x100$$

where A_s_, and A_c_ were the average absorbance of sample treated and control group respectively.

The assessment of nitric oxide (NO) synthesis or anti-proliferative activity was determined using the Griess method [28]. RAW 264.7 cells were plated in a 48-well plate and cultured for 24 h. Subsequently, encapsulated color pigment extracts doses (2–40 µg/mL) were administered to the cells alone and in combination with LPS (1 µg/mL) for 16 h. Nitrite production in the supernatant was analyzed using Griess reagent, and absorbance was measured at 595 nm. All the above treatments were performed in triplicate.

### 2.13 Development of functional gummies and its sensory analysis

Gummies were prepared by enriching natural encapsulated color pigment extracted from prickly pear and compared with commercially available synthetic color and further analyzed for visual analysis. Development of gummies was carried out according to following steps: First, sugar syrup was made by mixing and heating equal amount of refined sugar (30 g) and water (30 mL), till clear syrup. After getting clear syrup, citric acid (0.3 g) was added to make it invert sugar syrup. Further, equal amount of agar-agar (1.5 g) and pectin (1.5 g) were mixed thoroughly to get homogeneous mixture. Lastly, 1–2 drops of natural encapsulated or synthetic color were mixed to get uniform color. This gummy mixture was then poured in mold, cooled completely, and demolded for final product. Air bubbles were removed before gummy mixture poured in mold.

A semi-trained panel of three groups consisting of 20 students or employees each of the Food and Nutrition Biotechnology Lab performed the visual sensory evaluation. The evaluation was done using a nine-point hedonic scale, with the results being very dislike, dislike, acceptable, like, and very like. The goal of the visual sensory evaluation was to gain approval for natural color pigment over synthetic color, hence it was limited to color and visual acceptance.

### 2.14 Statistical analysis

All the analyses were carried out in triplicate and the results were provided as mean value with standard deviation. The obtained data were subjected to statistical analysis and the means compared by ANOVA Duncan’s new multiple range test (*p ≤ 0.05*) are presented.

## 3 Results and discussion

### 3.1 Response surface methodology (RSM): Fitting the model

**Fig. 2:**
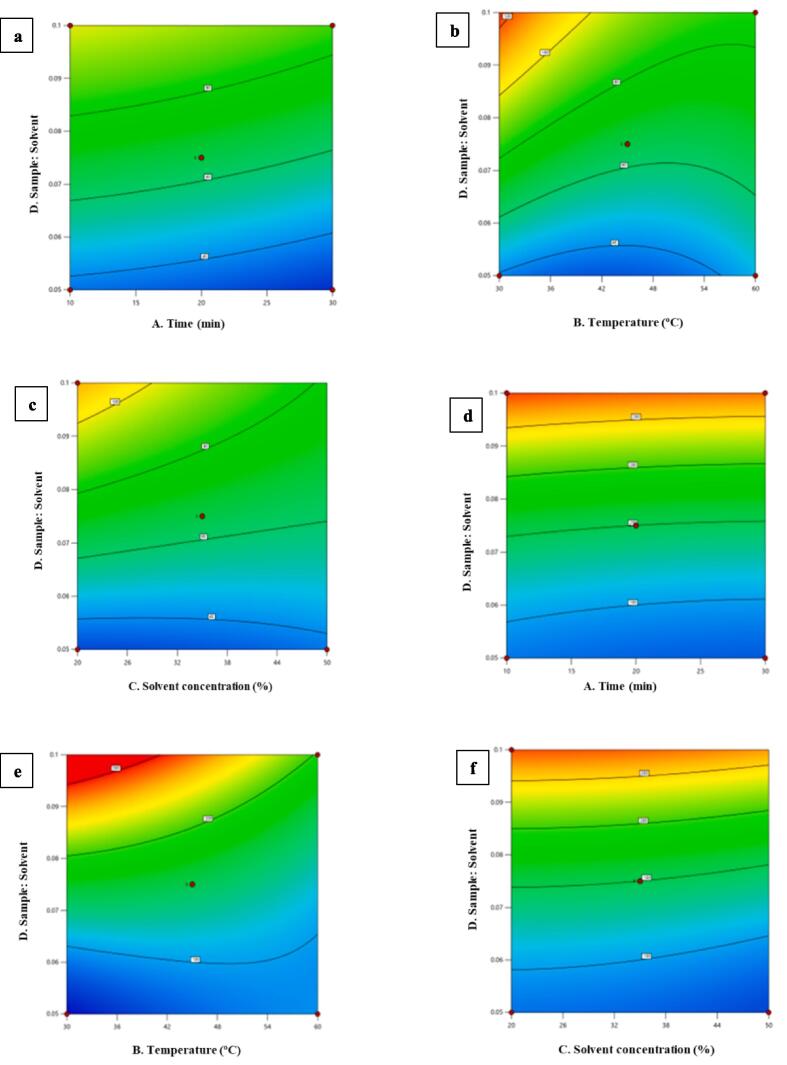
Graphs obtained by response surface methodology under different conditions for betaxanthin a) Solvent: sample-Time, b) Solvent: sample-Temperature, c) Solvent: sample-Solvent concentration; and for betacyanin d) Solvent: sample-Time, e) Solvent: sample-Temperature, f) Solvent: sample-Solvent concentration.

Response surface methodology (RSM) is a technique used to forecast how process parameters will interact and provide insight into how individual or combination process factors may affect overall process responses [28], [29]. Experiments were conducted using the Box-Behnken Design (BBD) method to determine the best combination for extracting the color pigment betalain from powdered prickly pear fruit. When determining recovery at a 95 % confidence interval, the ANOVA revealed that the RSM-suggested quadratic model for both betaxanthin and betacyanin was statistically significant (Table 2). To create a more straightforward and practical model, the insignificant terms (*p > 0.05*) were removed from the model using a backward elimination procedure. Lower p-values are generally thought to show each term's relative relevance. A lack of fit test was used to confirm the model's effectiveness. The results showed that the model had correctly matched the experimental data and were not statistically significant. In order to explain the interactions that take place between the independent variables, 3D surface plots are generated using the quadratic model equations (Fig. 2). These 3D plots allow a better understanding of the main and cross-product effects of the independent variables on-target responses. Plots of response surfaces illustrate how two elements interact while keeping the levels of the other factors constant. The purpose of these response surface plots was to quantify the ideal amount of each variable for maximal reaction and to comprehend how various variables interacted with one another.

**Table 2:** ANOVA table (p-value) for responses.

**Source**	**Betaxanthin**	**Betacyanin**
Significance	p-value	p-value
Model	*0.0019 (significant)*	*< 0.0001 (significant)*
A-Time	*0.1027*	*0.3411*
B-Temperature	*0.0473*	*0.0023*
C-Solvent Concentration	*0.1308*	*0.1946*
D-Sample: Solvent	*< 0.0001*	*< 0.0001*
AB	*0.5378*	*0.8709*
AC	*0.5241*	*0.4576*
AD	*0.9392*	*0.9457*
BC	*0.9454*	*0.9036*
BD	*0.1463*	*0.0025*
CD	*0.2240*	*0.9868*
A^2^	*0.8112*	*0.7722*
B^2^	*0.0515*	*0.2822*
C^2^	*0.7777*	*0.6396*
D^2^	*0.5823*	*0.0063*
Lack of Fit	0.0858 (not significant)	0.0679 (not significant)
RSM- coefficient of determination (*R^2^*)	0.9337	0.9521

### 3.2 Effect of process variables on extraction of betalain from prickly pear

The extraction of color pigment from prickly pear powder like betaxanthin and betacyanin was strongly affected by solvent to sample ratio (*p < 0.0001*) with the coefficient of determination (*R^2^* – 0.993 and 0.9521, respectively) of the obtained quadratic model as shown in Table 2. The quadratic model equations for every response are as follows:

**Betaxanthin:** 65.53 – 6.09A – 8.41B – 6.18C + 30.80D + 3.79AB + 3.92AC – 0.4650AD – 0.4175BC – 11.95BC – 9.86CD – 1.19A^2^ + 10.69 B^2^ + 1.43C^2^ – 3.03D^2^ **(1)**.

**Betacyanin:** 149.86 – 5.84A – 25.03B – 8.94C + 101.33D + 1.69AB + 7.84AC – 0.7100AD – 1.26BC – 49.95BC – 0.2223CD + 2.46A^2 –^ 9.52B^2^ – 4.06C^2^ + 30.29D^2^ **(2)**.

Here, A- Time; B- Temperature; C- Solvent concentration; D- Sample: Solvent.

It can be seen from equation 1 that betaxanthin extraction has negative correlation with time, temperature, and solvent concentration. There is decrease in betaxanthin content with an increase in treatment time, temperature given or solvent concentration. It can also be inferred from equation 1 that sample to solvent ratio has positive correlation with betaxanthin extraction. Moreover, betaxanthin response increased with increase in sample to solvent ratio which is statistically significant also (Table 2, Fig. 2a, 2b, and 2c). Similarly, equation 2 suggested negative correlation of betacyanin with time, temperature, and solvent concentration. Betacyanin has a positive correlation with sample to solvent ratio (Table 2, Fig. 2d, 2e, and 2f). Moreover, it can also be elucidated from Table 1 that both betaxanthin and betacyanin have strong statistical significance (*p < 0.0001*) of solvent to sample ratio and moderately statistical (*p < 0.05*) quadratic effect of extraction temperature (Table 2). There are few studies which corelates our study as well. According to the research published by Aalim et al. [30], we can observe from this study that the primary extraction parameters that affected the extraction of phenolic compounds were the solvent to sample ratio, extraction temperature, and time. Furthermore, temperature and solvent ratio have a big impact on how much anthocyanin can be extracted from black currants reported earlier [31]. Additionally, Ghasemzadeh et al. [32] investigated how several factors affected the antioxidant molecules that were extracted from red and brown rice bran.

### 3.3 Overall color analysis and encapsulation efficiency

**Fig. 3:**
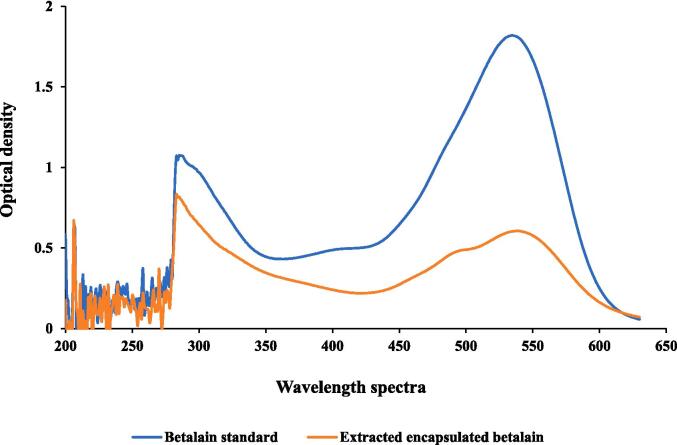
UV–visible spectra of encapsulated betalain from prickly pear and standard betalain.

The extracted color pigment has betaxanthin and betacyanin content of 294.53 and 563.75 mg/L, respectively, indicating overall betalain content (betaxanthin + betacyanin) to be 858.28 mg/L. Moreover, the color value was measured using hunter color lab having L value of 1.44 ± 0.23, a value of 6.37 ± 0.42 and b value of 2.15 ± 0.16. The UV-spectra of standard betalain and extracted betalain is given in Fig. 3, to confirm the visual reference. Furthermore, encapsulation of color pigment using glycerol is less explored. Encapsulation efficiency in glycerol-based color pigment was found to be 93.76 ± 0.77 %, indicating highly satisfactory encapsulation efficiency. Different encapsulation techniques have been applied on different fruit and vegetable juice which has been reported earlier. For example, Utpott et al. [33] reported encapsulation efficiency more than 90 % in powdered encapsulated red pitaya betalain color pigment. Similarly, encapsulation of cactus fruit by polysaccharide-proteins reported to have encapsulation efficiency more than 98 %, which is in powder form [34].

### 3.4 Thermal stability of extracted color pigments

**Fig. 4:**
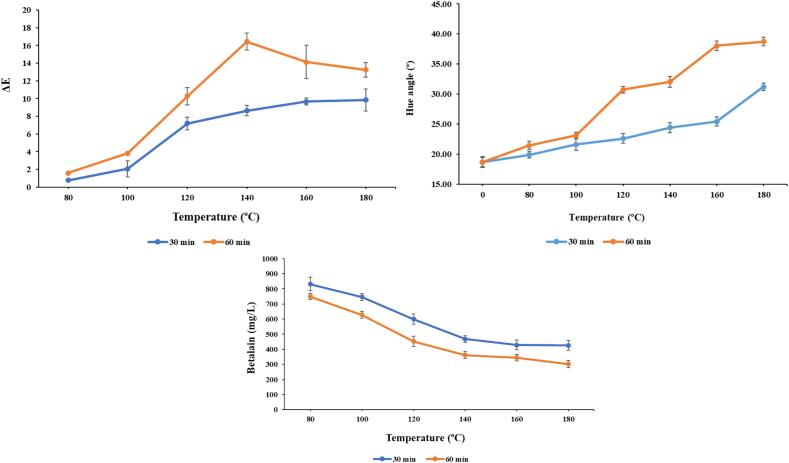
A) effect of thermal treatment on a) color difference (ΔE); b) Hue angle (°); c) Betalain content (mg/L) of encapsulated color pigment. Vertical bars depict standard error.

The stability of betalains as natural colorant significantly affects the overall perception of consumer experience. Therefore, it is necessary to expose the encapsulated color pigment to higher temperatures to evaluate its stability. As per procedure, the encapsulated color pigment was exposed to temperature varying from 80 °C to 180 °C for 30 and 60 min., respectively. In the present study, a gradual increase in color difference (ΔE) and slight shift in hue angle can be observed (Fig. 4a and b), indicating an effect on visual aspect of encapsulated color pigment. Moreover, a gradual decrease in the betalain content with in the increase in exposure time (30 and 60 min.) and temperature (80–180 °C) was observed (Fig. 4c). High temperature exposure (80 °C to 180 °C) increased the degradation rate of encapsulated color pigment which also proportionally indicated reduction of half-life (time required to reach concentration of pigment by 50 %) (Table 3). Furthermore, a significant decrease in degradation rate constant can be observed between 80 °C and 100 °C for 30 min. treatment but less gradual decrease has been observed in 60 min. treatment. This might be due to encapsulation by glycerol, protecting the overall color pigment over higher exposure of temperature. In other words, higher thermostability of glycerol helps in successfully encapsulating the color pigment, protecting it from degradation at higher temperature. López-Rubio and Lagaron [35] reported that glycerol-induced stabilization is uncertain, but it could be due to intrinsic viscosity and restricted molecular mobility. Similar kind of degradation in betalain (betaxanthin or betacyanin) content over exposure of heat treatment has been reported earlier. For example, Carreón‐Hidalgo et al. [36] reported degradation in overall betalain content from *Opuntia ficus-indica* on exposure of temperature range of 50–90 °C. Similarly, tomato lycopene, which is a hydrophobic compound, reported to be degraded over exposure to thermal treatment has been reported earlier [37]. Moreover, pomegranate anthocyanins, which are hydrophilic compounds, reported to be degraded by thermal treatment ranging from 50-95 °C (0–10 min.) [38].

**Table 3:** Betalain degradation rate constant (k value) and half-life estimation with elevated temperature for encapsulated betalain stability test.

**Temp. (°C)**	**k (30 min.)**	**t^1/2^ (min.)**	**k (60 min.)**	**t^1/2^ (min.)**
80	0.00046	1505.022^a^	0.000981342	706.176^a^
100	0.003667	188.992^b^	0.002268536	305.483^b^
120	0.005195	133.406^c^	0.004622318	149.925^c^
140	0.008792	78.821^d^	0.006253474	110.818^d^
160	0.010042	69.009^e^	0.006494958	106.698^e^
180	0.010151	68.2701802^f^	0.007547197	91.82217^f^

Different superscripted letters in the same column are significantly different (*p* < 0.05).

### 3.5 Degradation kinetics, predictive shelf-life, and stability of encapsulated color pigments

**Fig. 5:**
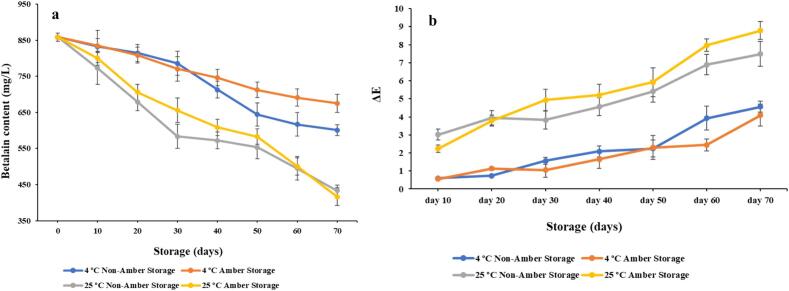
Effect of storage conditions on a) Betalain content (mg/L); b) Color difference (ΔE). Vertical bars depict standard error.

In order to observe changes in encapsulated color pigment during storage, it is necessary to have a thorough understanding of the factors that influence pigment degradation. For an objective evaluation of color deterioration in natural extracts, kinetic models are frequently employed [39], [40] In our study, 70-days stability of the betalain extracted from prickly pear was investigated, during storage under amber and non-amber conditions at two different storage temperatures (4 and 25 °C). As per Fig. 5a, ambient conditions (25 ± 2 °C) promoted the degradation of betalain pigment more as compared to refrigerated conditions (4 ± 2 °C). At 4 °C storage, betalain pigment stored at amber and non-amber conditions have 78.65 % and 69.99 % retention, respectively, at the end of 70th day. Whereas at 25 °C storage, pigment retention in amber and non-amber conditions at the end of 70th day was 48.47 % and 50.47 %, respectively. It is clearly observed from this data that amber storage at 4 °C has retained the maximum betalain contained followed by non-amber storage at 4 °C and non-amber storage at 25 °C (Fig. 5a). First, there is hardly any study which reported glycerol-based encapsulation of betalain color pigment. Second, the present study had one of the maximum retentions even after 70 days compared to recently reported encapsulated betalain studies. For example, Tekin et al. [41] reported 79.48 % betalain retention in capsules after 42 days storage. Also, Otálora et al. [42] reported 3–25 % retention of betalain pigment at the end of 25th day, considering storage at different humidity levels.

To develop a kinetic model that can predict colorant stability behavior, the rate of deterioration (k) for betalain under various storage circumstances was calculated. A first-order kinetic reaction model could satisfactorily explain the experimental data, as indicated by the correlation (*R^2^*), which varied between 0.95 and 0.97 (Table 4). Similar reports have been published earlier (21, 43). As per Table 4, degradation rate constant value (k) is more in 25 °C as compared to 4 °C stored pigments, though there was not significant difference between amber or non-amber storage at respective storage conditions. Betalain content can experience degradative reactions in high temperature, despite their structural stability, as reported in earlier report [36], [43], [44]. Similar kind of reports have been published earlier, where degradation of betalain happened during storage at higher temperature [21], [25]. Moreover, half-life of betalain pigment at 4 °C amber storage is 3 times higher than 25 °C amber storage. Overall, temperature was found to be the primary factor influencing the chemical stability of both betalains, regardless of light exposure [21], [46], [25]. Similarly, half-life of betalain pigment at 4 °C non-amber storage is 1.9 times higher than 25 °C non-amber storage. This effect could be explained by the fact that absorption of UV or visible light excites the pigment chromophore's π electrons to a more energetic state (π*), which either lowers the molecule's activation energy or increases its reactivity [45]. Overall, impact of storage temperature is significant as compared to its exposure to light.

**Table 4:** Degradation kinetics parameters [rate constant (k), half-life time (t_1/2_, days) of betalain during storage in the dark and normal light exposure, at different temperature (4, 25 °C) for 70 days.

**Storage conditions**	**k (days^−1^)**	**t_1/2_ (days)**	**Predictive shelf-life (days)**	***R^2^***
4 °C Non-Amber Storage	0.005097	135.9643^b^	271.9864^b^	0.96
4 °C Amber Storage	0.003429	202.0914^a^	404.2686^a^	0.97
25 °C Non-Amber Storage	0.009768	70.94418^c^	141.9185^c^	0.95
25 °C Amber Storage	0.010328	67.09804^d^	134.2246^d^	0.95

Different superscripted letters in the same column are significantly different (*p* < 0.05).

### 3.6 Effect of storage conditions on visual color of pigments

Visual color and its stability are an important prerequisite for consumer perception and its application in food products. In this part, the hunter color lab parameters were evaluated during a 70-day storage period under amber and non-amber conditions, at varying temperatures (4 and 25 °C), in order to explore the color characterization of the encapsulated betalain pigment extracts. In order to understand the color changes with sight of human perception, color difference (ΔE) was calculated. Fig. 5b depicts the color difference in chromatic properties and evolution of the betalain extracts under investigation. The variation in extracted color can be best described using ΔE, which is the combination of L*, a*, and b*, as recommended by the International Commission on Illumination (CIE): (i) minimal ΔE < 5, (ii) significant 5 ≤ ΔE < 12, and (iii) strikingly distinct ΔE ≥ 12 [46]. Regardless of amber or non-amber condition, storage of color pigment at 4 °C maintained a ΔE lower than the mentioned threshold value i.e., ΔE = 5 for 70 days, whereas pigment stored at 25 °C reached ΔE to 5 even at 40th day, which is quite satisfactory despite storage at room temperature (Fig. 5b). In summary, these findings demonstrate that temperature has a significant impact on ΔE variation during storage, while UV light shielding has no discernible effect on the evolution of either.

### 3.7 In-vitro digestibility

The bioactivity and bioavailability of potentially bioactive compounds are impacted during digestion because food ingredients are constantly exposed to physical (temperature), biochemical (pH), and chemical (enzymes) factors [47]. Understanding bio-accessibility is crucial for knowing its importance in relation to human health [48]. In this section, conventionally extracted color pigment was compared with encapsulated color pigment extracted according to current study. The betalain content of conventionally extracted color pigment before digestion was 1858.54 ± 78.5 mg/L whereas the encapsulated color pigment has betalain content of 858.28 mg/L, before digestion. The more betalain content in conventionally extracted color pigment was might be due to rotary evaporation process involved in it. Further after *in-vitro* digestion, conventionally extracted color pigment has betalain content of 19.17 mg/L, whereas encapsulated color pigment has betalain content of 17.57 mg/L. This clearly shows the effect of encapsulation as more in-vitro loss of betalain can be observed in conventionally extracted color pigment as compared to encapsulated color pigment. The bio-accessibility of betalain from conventionally extracted color pigment is 1.03 ± 0.09 % while encapsulated color pigment is 2.05 ± 0.03 %, which is twice the amount. Overall, encapsulation has helped the color pigment to pass the digestive system to reach the human system.

### 3.8 Cytotoxicity and anti-inflammatory properties

**Fig. 6:**
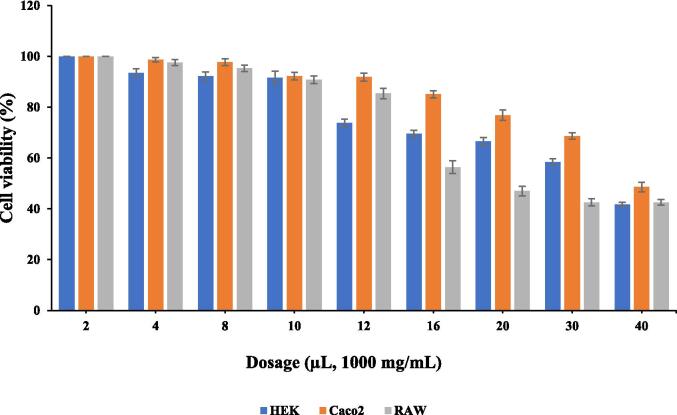
Cytotoxicity of encapsulated color pigment. Vertical bars depict standard error.

Recently, studies pertaining to betalains’ cell viability have drawn more attention. This is due to a rise in the inflammation disease in which an inflammatory process is connected to the generation of reactive oxygen species and degenerative illnesses as a result [49], [50]. The cell viability assay regarding RAW, HEK and cancer cells i. e. Caco2 cells is shown in Fig. 6. The cell viability assay showed no toxicity towards RAW, HEK and Caco2 cells when the concentration of color pigment extracts was in between 2–20 µL. No toxicity at lower concentration might be due to high concentration and viscosity of the sample (1000 mg/mL). Generally, color pigments are used in lower concentration and the encapsulated pigment is not toxic and suitable for food purpose. It has been earlier reported that betalains exhibit biological actions that include scavenging free radicals and protecting against various disorders [51], [52].

**Fig 7:**
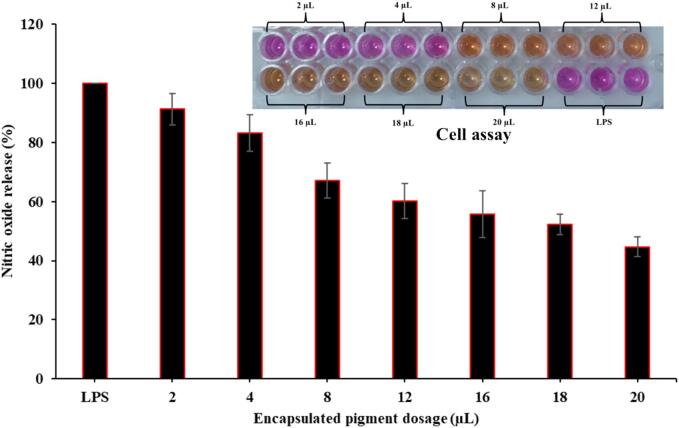
Anti-inflammatory activity of encapsulated color pigment. Vertical bars depict standard error. LPS represents lipo-polysaccharide from macrophagic cells.

Anti-inflammatory properties responses of extracted and encapsulated betalain pigment were analyzed against macrophages (Fig. 7). In the analysis, lipopolysaccharide (LPS) was treated with encapsulated betalain pigment at the concentration of 2 to 20 µL, further observing production of nitric oxide (NO) with increase in concentration of pigment treatment. For the Fig. 6b, it can be clearly seen that significant reduction was found in NO release as the dosage of encapsulated pigment increases (2–20 µL). The results are in accordance with previously published studies [49], [52].

### 3.9 Visual sensory analysis of functional gummies

**Fig. 8:**
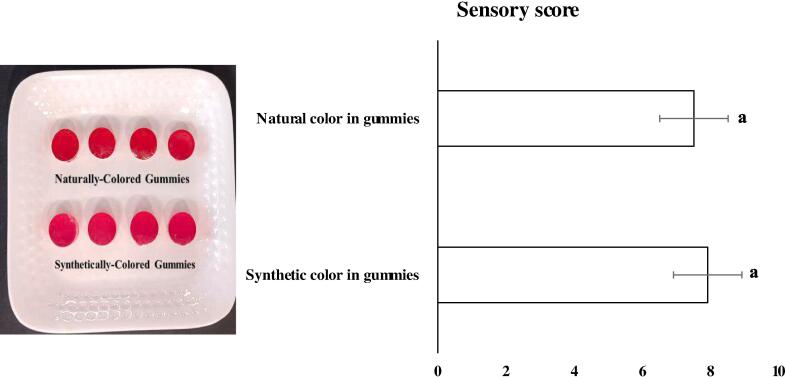
Visual sensory acceptability of encapsulated betalain (natural color extracted from prickly pear) incorporated gummies in comparison of synthetic color (Carmoisine) incorporated gummies. Vertical bars depict standard error. Similar superscripted letter ‘a’ upon column represents non-significant difference (*p* < 0.05).

A key indicator for deciding whether an ingredient is appropriate to fortify a particular food depends upon its sensory evaluation. Thus, this visual sensory test can be viewed as an indicator of consumers' acceptance of the finished product. Sensory score of gummies incorporated with natural and synthetic color pigment is shown in Fig. 8. It can be clearly seen that there is no significant difference between sensory score of both type of gummies, indicating visual acceptance of encapsulated natural color pigment incorporated in gummies. According to previously published report, betalain-rich capsules produced by ionic gelation with calcium alginate from a betalain-rich extract of *Opuntia ficus indica* fruit were included into gummy candies as a model food system [42].

## 4 Conclusion

In this study, we focused on ultrasound-assisted extraction and encapsulation of betalains from an underutilized, nutritionally, and medicinally valuable fruit called prickly pear, highlighting its significant advancement in the field of natural color pigment extraction. This research meticulously optimized the extraction process with the optimum yield of betalains having biological properties. Encapsulation using a greener solvent like glycerol is a novel method to improve the bio-accessibility of the betalains, showing twice the accessibility in comparison to conventionally extracted color pigments. Moreover, the stability study of encapsulated betalains ensures the longevity of visual color parameters and bioactive properties under various conditions such as storage at 4 and 25 °C, with or without amber conditions.

The predictive shelf-life of encapsulated betalains ranges from 4 months to 12 months depending on storage conditions, which is highly significant for a natural compound. Additionally, the encapsulated color pigment has shown anti-inflammatory properties and no cytotoxicity in vitro, making it suitable for food products like functional gummies. These may be considered as possible alternative candidates for market-available gummies with artificially added colors, which have various side effects on human health. Thus, the findings of this study have far-reaching implications for the extraction and encapsulation of natural color compounds and further incorporating it into development of other functional foods which can significantly contribute to the field of nutraceuticals and food science.

## CRediT authorship contribution statement

**Deepak Mehta:** Resources, Methodology, Formal analysis. **Kritika Kuksal:** Investigation, Formal analysis, Data curation. **Kamlendra Yadav:** Validation, Resources, Methodology. **Sudesh Kumar Yadav:** Writing – review & editing, Data curation. **Yuqin Zhang:** Software, Visualization. **Shivraj Hariram Nile:** Writing – review & editing, Writing – original draft, Supervision, Funding acquisition, Conceptualization.

## Declaration of competing interest

The authors declare that they have no known competing financial interests or personal relationships that could have appeared to influence the work reported in this paper.
